# Effect of *Piper betle* and *Brucea javanica* on the Differential Expression of Hyphal Wall Protein (*HWP1*) in Non-*Candida albicans Candida* (NCAC) Species

**DOI:** 10.1155/2013/397268

**Published:** 2013-06-18

**Authors:** Wan Himratul Aznita Wan Harun, Nur Alyaa Jamil, Nor Hazwani Jamaludin, Mohd-Al-Faisal Nordin

**Affiliations:** Department of Oral Biology, Faculty of Dentistry, University of Malaya, 50603 Kuala Lumpur, Malaysia

## Abstract

The study aimed to identify the *HWP1* gene in non-*Candida albicans Candida* species and the differential expression of *HWP1* following treatment with *Piper betle* and *Brucea javanica* aqueous extracts. All candidal suspensions were standardized to 1 × 10^6^ cells/mL. The suspension was incubated overnight at 37 °C (*C. parapsilosis*, 35°C). Candidal cells were treated with each respective extract at 1, 3, and 6 mg/mL for 24 h. The total RNA was extracted and reverse transcription-polymerase chain reaction was carried out with a specific primer of *HWP1*. *HWP1* mRNAs were only detected in *C. albicans*, *C. parapsilosis*, and *C. tropicalis*. Exposing the cells to the aqueous extracts has affected the expression of *HWP1* transcripts. *C. albicans*, *C. parapsilosis*, and *C. tropicalis* have demonstrated different intensity of mRNA. Compared to *P. betle*, *B. javanica* demonstrated a higher suppression on the transcript levels of *HWP1* in all samples. *HWP1* was not detected in *C. albicans* following the treatment of *B. javanica* at 1 mg/mL. In contrast, *C. parapsilosis* and *C. tropicalis* were shown to have *HWP1* regulation. However, the expression levels were reduced upon the addition of higher concentration of *B. javanica* extract. *P. betle* and *B. javanica* have potential to be developed as oral health product.

## 1. Introduction

*Candida* is a genus of yeast-like fungi that are commonly part of the normal flora of the mouth, skin, intestinal tract, and vagina. Areas of recovery of *Candida *species in the oral cavity include the dentition, tongue, cheeks, and palatal mucosa, as well as from restorative materials and prostheses [1]. Morphologically, the size of *Candida* cell is about 4–6 *μ*m. Approximately, around 196–200 species of *Candida* have been identified so far [2]. Oral candidiasis (oral thrush) is a common candidal infection in human oral cavity. Among the several *Candida *species, *Candida albicans *is most frequently isolated from patients with candidiasis. *C. albicans *has been reported to have the ability to grow as yeast, hyphae, and pseudohyphae [3]. However, recently there are reports on the prevalence of non*-Candida albicans Candida *(NCAC) species such as *Candida dubliniensis*,* Candida glabrata*,* Candida krusei*,* Candida lusitaniae*,* Candida parapsilosis, *and *Candida tropicalis* being associated with oral candidiasis [4, 5].

The opportunistic characteristic of *Candida *species allows the transition from harmless to a pathogenic microorganism and is responsible for a wide range of systemic and superficial infections in the immunocompromised hosts [6]. A number of virulence factors such as adhesion, hydrolytic enzyme production for example, proteinases and phospholipases, hyphal formation, and phenotypic switching may be involved to establish an infective process. 

Candidal cell wall is a dynamic structure and represents the prime site to interact with oral epithelial cells. *HWP1* is the first cell surface protein known to be required for *C. albicans* biofilm formation *in vivo *[7]. The *HWP1* functions as adhesins in the cell wall, promoting the attachment of candidal cells to host mucosal surface. Although it has been described as hypha-specific adhesins [8], recent studies have shown that *HWP1 *transcript may arise from pseudohyphal growth forms [9]. 

Malaysia is well known for its plant biodiversity. Some of them are utilized for medicinal purposes. *Brucea javanica* (L.) Merr is a member of the family Simaroubaceae. The seeds of this plant have been used in traditional medicine of Indonesia and China [10, 11]. It has also been reported to exhibit anticancer property [12] and used as an insecticide to treat malaria and amoebic dysentery [13]. Another common plant which is used as remedy by the Asian folks is *Piper betle* L. (Piperaceae). The *P. betle *leaves have long been used in the Indian local system of medicine for its antioxidant and antimicrobial properties [14, 15]. It is also popular as an antiseptic and is commonly applied on wounds and lesions for healing effects [16, 17]. Due to its strong pungent aromatic flavour, it is widely used as a postmeal mouth freshener and masticatory by the Asian people. This particular property has paved way for further experimental studies on *P. betle* [18]. 

In the present study, we focused on the association of *HWP1* in the NCAC species. *HWP1* of *C. albicans *has been extensively studied, reflecting the growing concern over its role in various stages of infections. Information on the expression levels of *HWP1* transcripts that encode adhesins amongst the NCAC species, however, is scarce. The hyphal formation between *Candida *species has the propensity to express distinctive pattern of *HWP1*. Therefore, this study aimed to identify the expression of *HWP1 *mRNA transcript in NCAC species and to examine the regulation of *HWP1 *following treatment with *B. javanica *and *P. betle* aqueous extracts.

## 2. Materials and Methods

### 2.1. Preparation of Crude Aqueous Extract

Fresh leaves of *Piper betle *and the seeds of *Brucea javanica *were collected from local areas in Brickfields and Sekinchan, Malaysia. Crude aqueous extracts of the plants were prepared [19]. Each specimens was weighted at 100 g, followed by rinsing under running tap water, and dried for 2 days at 60°C. The respective dried specimen was homogenized in distilled water at a ratio of specimen to water of 1 : 10. The homogenate was heated at high temperature and concentrated to 1/3 of the original volume. The decoction was filtered through a Whatman No. 1 filter paper. The filtrate was freeze-dried (EYELA FDU-1200, Tokyo) overnight, and the fine powder obtained was kept in a sterile Falcon tube and stored at 4°C.

### 2.2. Preparation of Candidal Suspension

Seven candidal strains, *Candida albicans* (ATCC 14053), *Candida dubliniensis* (ATCC MYA-2975), *Candida glabrata* (ATCC 90030), *Candida krusei* (ATCC 14243), *Candida lusitaniae* (ATCC 64125), *Candida parapsilosis* (ATCC 22019),d and *Candida tropicalis* (ATCC 13803), were purchased from the American Type Culture Collection (ATCC), USA. The candidal cells were revived in 10 mL Yeast Peptone Dextrose (YPD) broth and the turbidity of the suspension was adjusted spectrophotometrically to an optical density (OD_550 nm_) of 0.144 which is equivalent to 1 × 10^6^ cells/mL. This standard suspension was used throughout the experiment.

### 2.3. Treatments of *Piper betle* and *Brucea javanica* on *Candida* Species

The standardised suspension (10^6^ cells/mL) of each strain was incubated overnight at 37°C (*C. parapsilosis*, 35°C) to allow the propagation of cells. The cultures were acclimatized for 1 h, followed by the addition of sub-MICs of 1, 3, and 6 mg/mL. The MICs of *Piper betle *and *B. javanica *were determined between 25 to 12.5 mg/mL [19]. After 24 h, 3 mL of each respective culture was centrifuged at 8,000 ×g (4°C) for 5 min and the supernatant was discarded. The treated pellets were washed twice with phosphate-buffered saline (PBS, pH 7.2), and the total RNA extraction was performed. For the untreated samples, total RNA was isolated from the nontreated pellets. 

### 2.4. Oligonucleotide Sequences

A pair of primer specifically designed for *HWP1 *mRNA was purchased from the 1st BASE Laboratories, Malaysia. The primer was used in reverse transcription-polymerase chain reaction (RT-PCR) to amplify the sequences that encode for *HWP1* in *Candida *species. *ACT1* primer was used as a positive control to detect *Candida *species carrying *HWP1* gene. 

### 2.5. Optimization of Primers

Annealing temperature is a key factor in performing PCR amplification for reaction specificity. The optimization was carried out with the Mastercycler gradient. A gradient of 60°C ± 5°C was programmed and the temperature distribution in the individual columns of the block was set at 55°C, 57°C, 59°C, 61°C, 63°C, and 65°C.

### 2.6. Total RNA Extraction

Freshly prepared candidal suspensions were inoculated into 10 mL YPD broth and incubated overnight at 37°C in a rotary shaking incubator. 3 mL of the respective cell suspension was centrifuged at 2,000 ×g for 10 min in a 4°C refrigerated centrifuge and the supernatant was discarded. The pellet formed was washed with sterile PBS, pH 7.2. Following that, total RNA was extracted using an easy-RED BYF Total RNA Extraction kit (Intron Biotechnology Inc.) according the manufacturer's instruction. 

Next, 250 *μ*L of prelysis buffer was added and re-suspended thoroughly. A 750 *μ*L of easy-RED solution was added, vigorously mixed for 15 s, and left at room temperature for 5 min. Following the addition of 200 *μ*L of chloroform, the samples were vigorously mixed for 15 s and left at room temperature for 5 min. 

The samples were centrifuged at 8,000 ×g (4°C)  for 15 min, and the colourless aqueous phase formed was transferred to a new microcentrifuge tube. An equal volume of isopropanol (2-propanol) was added, mixed by inverting the tube for 6-7 times, and left at room temperature for 10 min. The suspension was recentrifuged at 8,000 ×g (4°C) for 10 min, and the supernatant was discarded without disturbing the pellet. 

One millilitre of 70% ethanol was added and mixed by inverting the tube several times. The mixture was centrifuged at 8,000 ×g (4°C) for 5 min. The supernatant was discarded and the RNA pellet was left to dry. The pellet was dissolved in 25 *μ*L of RNase-free water and stored at −80°C.

### 2.7. RNA Quantification

The RNA samples were analyzed for its integrity using the Agilent 2100 Bioanalyzer and all samples were considered to have high quality RNA by referring to the RIN number [22]. 

### 2.8. Reverse Transcription-Polymerase Chain Reaction

Fifty ng/mL of the RNA template and 1 mL of each primer were mixed in 10 *μ*L of *Prime *RT-PCR Premix 2X (GENET BIO) which contained *HS Prime Taq *DNA polymerase, *Prime MMLV *reverse transcriptase, reaction buffer, 0.1 mM dNTPs mixture, RNase inhibitor, protein stabilizer, and enhancers for cDNA synthesis. RNase-free water was added up to a total reaction volume of 20 *μ*L.

Reverse transcription was carried out at 42°C for 30 min to synthesis the cDNA, followed by denaturation at 94°C for 10 min to deactivate the reverse transcriptase and activate the *HS Prime Taq *DNA polymerase. The samples were subjected to 30 cycles of denaturation (94°C), annealing (56°C to 64°C), and extension (72°C), each for 30 s. Lastly, the final extension was at 72°C for 5 min. 

The amplicon of 6 *μ*L was separated by electrophoresis in 1.5% (w/v) of agarose gel and stained with ethidium bromide. A Tris-borate-EDTA (TBE) was used as a running buffer and 100 bp DNA ladder (BIO-RAD) was used as a molecular weight marker. The expression of the gene was visualized by ultraviolet (UV) illumination (Alphaimager 2200, Alpha Innotech). 

## 3. Results 

**Figure 1:**
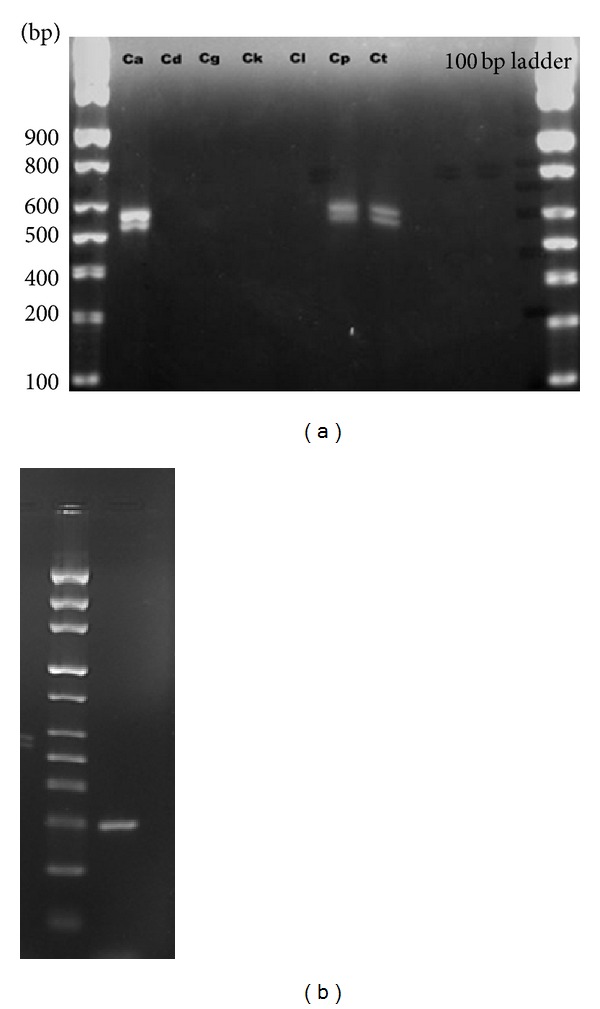
(a) Detection of *HWP1 *mRNAs from seven *Candida *species (Ca: *C. albicans*, Cd: *C. dubliniensis*, Cg: *C. glabrata*, Ck: *C. krusei*, Cl: *C*. *lusitaniae*, and Cp: *C*. *parapsilosis*, Ct: *C*. *tropicalis*) cultured in normal growth condition. (b) *ACT1 *gene (positive control).

The expression of *HWP1 *transcript was investigated in seven *Candida *species cultured in YPD broth for 24 h. mRNAs from all candidal spp. were loaded accordingly and results of gel electrophoresis after RT-PCR are shown in Figure 1. While *ACT1 *was used as the positive control, it was observed that *HWP1 *mRNAs were only expressed in *C. albicans, C. parapsilosis*,* and C. tropicalis *after three different independent experiments. No *HWP1 *transcripts were detected in *C. dubliniensis*, *C. glabrata*, *C. krusei*, and *C. lusitaniae *(Figure 1).

**Figure 2:**
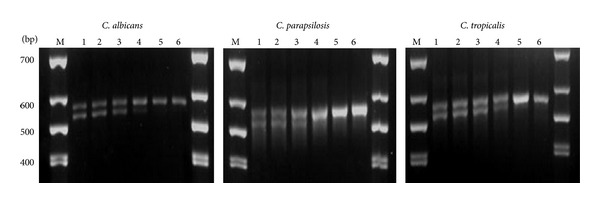
Detection of *HWP1 *mRNAs from *C. albicans*, *C. parapsilosis*, and *C. tropicalis* cultured in normal growth condition. PCR optimization (55 to 65°C) was performed. Lane 1 (55°C), Lane 2 (57°C), Lane 3 (59°C), Lane 4 (61°C), Lane 5 (63°C), and Lane 6 (65°C).

Double DNA bands were seen following agarose gel electrophoresis, indicating the nonspecific products. Thus, optimization of the PCR annealing temperature was carried out to determine the suitable temperature for specific binding of *HWP1* primer. Following optimization, it was found that the specific primer of *HWP1 *required an ideal temperature of between 61°C to 63°C in order to obtain a specific product of 572 bp (Figure 2).

**Figure 3:**
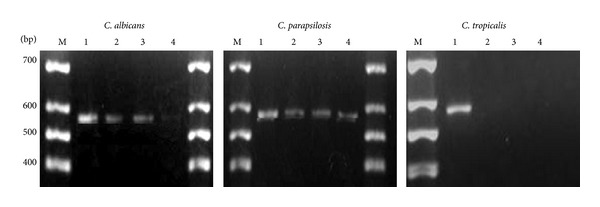
Expression of *HWP1 *mRNAs in *C. albicans*, *C. parapsilosis*, and *C. tropicalis *following treatment of *P. betle *at sub-MICs of 1, 3 and 6 mg/mL. Untreated *Candida *spesies (Lane 1), 1 mg/mL (Lane 2), 3 mg/mL (Lane 3), and 6 mg/mL (Lane 4).

Our study has found that exposing the candidal cells to the aqueous extracts of *P. betle *and *B. javanica *apparently has affected the expression of *HWP1 *transcripts with increased extract concentrations. *C. albicans*, *C. parapsilosis*d and *C. tropicalis* have demonstrated different intensity of mRNA which was observed in the agarose gel.  Figure 3 shows that the expression of *HWP1 *in *C. albicans *was reduced in a dose-dependent manner as higher concentration of *P. betle* extract used. At 6 mg/mL the band is hardly seen. *C. tropicalis *was observed not expressing *HWP1 *following exposure to 1, 3, and 6 mg/mL of *P. betle* extract.

**Figure 4:**
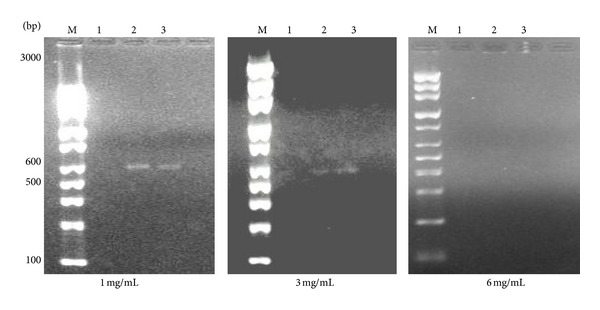
Expression of *HWP1 *mRNAs in *C*. *albicans *(Lane 1), *C*. *parapsilosis* (Lane 2), and *C*. *tropicalis *(Lane 3) following treatment of *B. javanica *at sub-MICs of 1, 3, and 6 mg/mL.

Compared to *P. betle *treatment, *B. javanica *demonstrated a higher suppression on the transcript levels of *HWP1 *in *C. albicans*, *C. parapsilosis*,and *C. tropicalis *(Figure 4). It was shown that *HWP1* was not detected in *C. albicans *following treatment of *B. javanica *even at 1 mg/mL. In contrast, *C. parapsilosis* and *C. tropicalis *were shown to have *HWP1 *regulation. However, the expression levels were reduced upon the addition of higher concentration of *B. javanica* extract. The *HWP1 *transcripts of *C. albicans*, *C. parapsilosis*, and *C. tropicalis *were not detected in *B. javanica* treated samples of 6 mg/mL. Findings suggest the transcription levels of *HWP1 *in *Candida *species may be suppressed by the addition of the extracts and were very much dependent on its environment.

## 4. Discussion

 Most microorganisms including *Candida *species have different mechanisms to adhere and invade the mucosal tissues in order to sustain their existence in the oral cavity. Several metabolic pathways or interactions could be involved between the cells and the host. Gene expression is known as the fundamental characteristic to understand the cellular functions which contribute to oral infection. Our study focuses on *HWP1 *gene that code for the adhesins-associated protein in the cell wall. *ACT1 *was only used as the positive control to detect *Candida *species carrying this specific gene. Hyphal growth forms of *Candida albicans* have been reported to be abundantly coated with an adhesin denoted *HWP1* (Hyphal Wall Protein 1) [23]. It has been studied in *C. albicans *but there is lack of information of its presence and expression in NCAC species [23]. Unlike other microbial adhesins which adhere through hydrophobic or lectin-like interactions, *HWP1 *forms covalent attachments to proteins on human buccal epithelial cells in host tissue [24]. *HWP1* is also required as first cell surface protein *in vivo *for biofilm formation [7]. Furthermore, this is the first study to investigate the potential of two plant extracts—*Piper betle *and *Brucea javanica*—specifically on the regulation of *HWP1*. These two plant extracts have been reported to exhibit antifungal effect on seven oral *Candida* species [25]. 

In normal growth condition where sufficient nutrients are provided, *HWP1 *mRNA transcripts were positively regulated in *C. albicans*, *C. parapsilosis*, and *C. tropicalis*. The expression of *HWP1* in these three *Candida *species indicates the ability of the cells to produce adhesins that are covalently linked to the cell wall glucan through a remnant of its glycosylphosphatidylinositol (GPI) anchor, leading to cell adhesion and biofilms formation [26]. The open reading frames (ORFs) that contain the region coding for *HWP1 *can be detected by the primers (Table 1) were which originally designed for *C. albicans*. This suggests that *C. parapsilosis *and *C. tropicalis *are possibly sharing the identical sequence of *HWP1 *with *C. albicans.* This is contrary to a study which previously reported that *HWP1 *was expressed only in *C. albicans* [23].

Although *C. albicans *and *C. dubliniensis *have a close phylogenetic relationship, no *HWP1 *transcript was detected and both species seems to have distinct capacity of the cell wall protein [23]. There are a few studies which showed that *C. dubliniensis *forms fewer true hyphae than *C. albicans* [27, 28], and the lack of *HWP1 *adhesins being produced may partly account for its reduced capacity to adhere and less able to establish systemic infection. This is in agreement with a recent study showed that the adhering ability of *C. dubliniensis *to salivary pellicle was less compared to *C. albicans *[25].

*C. glabrata *and *C. albicans *have the basic cell wall structure in common but displayed very distinct features ranging from the presence or absence of certain surface proteins. Unlike *C. albicans*, it is more dependent on lectins (*EPA *gene products) and exists as a multilayer structure of the yeast form [29]. Therefore, *C. glabrata *does not rely on the production of *HWP1* to establish an infection.

 Findings showed that the expression of *HWP1 *transcripts was drastically reduced following treatment with the extracts. The addition of extracts probably has created environmental stress and demonstrated fungistatic effect on the cells. The uptake of ions and nutrients which depend on the integrity of the cell wall may be restricted. Several regulatory elements as well as the transcription of respective genes may be deactivated while waiting for the environment to be adequate for growth. This could lessen the chance for the cells to propagate and thus inhibits the hyphal formation. This could possibly explain that the transcription of *HWP1 *was suppressed. The low expression of *HWP1 *indicates that the level of adhesins was affected by the extracts. The lack of *HWP1* being produced will distort the virulence trait of the candidal cells. 

## 5. Conclusion

The expression of *HWP1 *transcript may indicate the production of adhesins in *C. albicans*, *C. parapsilosis*, and *C. tropicalis*. The transcript levels were affected following treatments with *P. betle *and *B. javanica *extracts, suggesting that the level of adhesins being produced by the candidal cells may be lacking. Subsequently, the integrity of the cell wall will be compromised and the adhesion progress will be interrupted. Findings concluded that *P. betel* and *B. javanica* aqueous extracts have a great potential to be developed as oral health care product. 

## Figures and Tables

**Table 1:** Oligonucleotide sequences of *HWP1* and *ACT1*.

Primers^a^	Sequences (5′ → 3′)^b^	Temp (°C)	bp^c^
Forward (*HWP1*_For)	CCATGTGATGATTACCCACA	60.9	572
Reverse (*HWP1*_Rev)	GCTGGAACAGAAGATTCAGG	61.7	
Forward (*ACTI*_For)	GGCTGGTAGAGACTTGACCAACCATTTG	67.6	304
Reverse (*ACT1*_Rev)	GGAGTTGAAAGTGGTTTGGTCAATAC	61.4	

^a^Primer templates are chosen as prescribed in Naglik et al. [20] and Tavanti et al. [21].

^b^Melting temperatures are analysed using Oligo Analyzer 1.2.

^c^RT-PCR product size (bp, base pair).
